# Resting-state fMRI can detect alterations in seizure onset and spread regions in patients with non-lesional epilepsy: a pilot study

**DOI:** 10.3389/fnimg.2023.1109546

**Published:** 2023-05-05

**Authors:** Anish V. Sathe, Caio M. Matias, Michael Kogan, Isaiah Ailes, Mashaal Syed, KiChang Kang, Jingya Miao, Kiran Talekar, Scott Faro, Feroze B. Mohamed, Joseph Tracy, Ashwini Sharan, Mahdi Alizadeh

**Affiliations:** ^1^Department of Neurological Surgery, Thomas Jefferson University, Philadelphia, PA, United States; ^2^Department of Neurological Surgery, University of New Mexico, Albuquerque, NM, United States; ^3^Department of Radiology, Thomas Jefferson University, Philadelphia, PA, United States; ^4^Department of Neurology, Thomas Jefferson University, Philadelphia, PA, United States

**Keywords:** epilepsy, functional connectivity, resting-state, non-lesional, neuroimaging

## Abstract

**Introduction:**

Epilepsy is defined as non-lesional (NLE) when a lesion cannot be localized via standard neuroimaging. NLE is known to have a poor response to surgery. Stereotactic electroencephalography (sEEG) can detect functional connectivity (FC) between zones of seizure onset (OZ) and early (ESZ) and late (LSZ) spread. We examined whether resting-state fMRI (rsfMRI) can detect FC alterations in NLE to see whether noninvasive imaging techniques can localize areas of seizure propagation to potentially target for intervention.

**Methods:**

This is a retrospective study of 8 patients with refractory NLE who underwent sEEG electrode implantation and 10 controls. The OZ, ESZ, and LSZ were identified by generating regions around sEEG contacts that recorded seizure activity. Amplitude synchronization analysis was used to detect the correlation of the OZ to the ESZ. This was also done using the OZ and ESZ of each NLE patient for each control. Patients with NLE were compared to controls individually using Wilcoxon tests and as a group using Mann-Whitney tests. Amplitude of low-frequency fluctuations (ALFF), fractional ALFF (fALFF), regional homogeneity (ReHo), degree of centrality (DoC), and voxel-mirrored homotopic connectivity (VMHC) were calculated as the difference between NLE and controls and compared between the OZ and ESZ and to zero. A general linear model was used with age as a covariate with Bonferroni correction for multiple comparisons.

**Results:**

Five out of 8 patients with NLE showed decreased correlations from the OZ to the ESZ. Group analysis showed patients with NLE had lower connectivity with the ESZ. Patients with NLE showed higher fALFF and ReHo in the OZ but not the ESZ, and higher DoC in the OZ and ESZ. Our results indicate that patients with NLE show high levels of activity but dysfunctional connections in seizure-related areas.

**Discussion:**

rsfMRI analysis showed decreased connectivity directly between seizure-related areas, while FC metric analysis revealed increases in local and global connectivity in seizure-related areas. FC analysis of rsfMRI can detect functional disruption that may expose the pathophysiology underlying NLE.

## 1. Introduction

Epilepsy is a disease of abnormal connections in the brain (Engel et al., 2013). Seizure generation reflects abnormal synchronous firing of neurons (Fisher et al., 2005). Focal epilepsy is the most common form of epilepsy, a disease which may affect up to 1% of the general population (Sander and Shorvon, 1996). Seizures in focal epilepsy have been found to spread via connections between the region of seizure onset and other brain regions, such as the thalamus (Bertram, 2014). Abnormal networks in the brain may facilitate the spread of seizures and may be involved in the manifestation of clinical signs and symptoms seen in epilepsy (Kramer and Cash, 2012). Pathologic changes in brain networks may underlie these alterations in connectivity.

Currently, stereotactic electroencephalography (sEEG) can be used to detect regions of seizure onset and seizure spread (Gupta et al., 2020). fMRI has been shown to be a promising modality to detect similar measures of functional synchronization and correlations to sEEG and guide epilepsy treatment, such as surgery (Winston, 2015; Su et al., 2019).

Fluctuations in blood-oxygen-level-dependent (BOLD) signal over a time course can be detected by fMRI. This signal corresponds to the relative state of oxygenated and deoxygenated hemoglobin in each region of the brain, with higher BOLD signal serving as a proxy for increased neuronal activation (Logothetis, 2003). Temporal correlations of BOLD signal fluctuations between different brain regions have been shown to represent functional synchronization between the areas (Biswal et al., 1995). Functional synchronization can be represented through the nondirectional synchronization between different regions (functional connectivity, FC), or through directional effects of one region on the other (effective connectivity, EC) (Stephan and Friston, 2010). In other words, FC signifies the degree of synchronization between different brain regions, while EC provides mechanistic information of the causal effect of one brain region on another. BOLD signal fluctuations at rest represent spontaneous neuronal activation and may be used to define resting-state FC in the brain (Fox and Raichle, 2007).

Clinical neuroimaging is often used to detect structural lesions that may represent the focus of the seizure onset to derive prognostic information and plan for surgical intervention. Although there is no global standard for such epilepsy workup, the process typically includes different modalities of MRI (such as T1, T2, and FLAIR), and other modalities such as PET and SPECT (Pardoe and Kuzniecky, 2014). In addition, video EEG monitoring, neuropsychological evaluation, and seizure semiology are tools used during the epilepsy surgery work-up (Chassoux et al., 2010; Tufenkjian and Lüders, 2012; Maganti and Rutecki, 2013; Feng et al., 2014; Fernandez-Baca Vaca et al., 2018; Mayoral et al., 2019; Hasan and Tatum, 2021; Primiani et al., 2021). However, a subset of patients may not show an obvious lesion acting as the epileptogenic zone through these imaging modalities. These cases can be grouped under the term “non-lesional epilepsy (NLE).” The lack of identification of specific structural abnormalities can be due to a multitude of reasons, including the seizure onset zone arising from diffuse tissue abnormalities or pathologies too subtle to be detected by standard neuroimaging (Pardoe and Kuzniecky, 2014). Stereotactic electroencephalography (sEEG) is a useful method to help localize the epileptogenic zone in patients with NLE through the implantation of electrodes to detect epileptic signal spikes (Bartolomei et al., 2017). Clinically, distinguishing lesional epilepsy from NLE is important as NLE has shown to have much lower response rates to surgical management (Cascino et al., 1992; Téllez-Zenteno et al., 2010). There is much work being done to increase the effectiveness of MRI in localizing lesions in NLE. One such method is the utilization of simultaneous EEG-fMRI recordings, in which ictal EEG spikes are correlated with BOLD signal spikes on fMRI (Pittau et al., 2012a; Pardoe and Kuzniecky, 2014). More recent methods include the use of powerful 7T MRI machines with higher resolutions than conventional 1.5T or 3T machines, allowing for the detection of more detailed anatomical aberrations (Park et al., 2021). However, despite improved localization abilities, such modalities may still be unable to detect lesions in up to 35-40% of patients (Pittau et al., 2012a; Feldman et al., 2019; Park et al., 2021). Regardless, prognostic factors including the localization of abnormal patterns via functional imaging such as fMRI and the concordance of MRI patterns and EEG localization have shown to be correlated to better surgical outcomes (Jack et al., 1994; Bell et al., 2009; Zuo et al., 2010).

Due to the implications of functional imaging in detecting difficult-to-find lesions in epilepsy, fMRI analysis holds potential in improving treatment of patients with NLE. There are many different methods of BOLD signal analysis using fMRI to produce functional information. Seed-to-voxel analysis uses the average BOLD signal waveform over a time course from one “seed” region and detects synchronization with every other voxel in the fMRI scan. Amplitude of low-frequency fluctuations (ALFF) represents the mean of amplitudes within a specific frequency range from a fast Fourier transform of the specific voxel's time course (Yang et al., 2007; Wang et al., 2019). Fractional ALFF (fALFF) is a normalized version of ALFF which accounts for the relative contribution of specific waveforms in the entire frequency range that was analyzed. These two metrics represent the low-frequency signal fluctuations and therefore provide valuable information on fMRI signal. Regional homogeneity (ReHo) is based on a rank-based Kendall's coefficient of concordance that compares the synchronization of signal fluctuation over the time course of interest in a voxel and the 26 voxels neighboring it (Zang et al., 2004). This metric describes the relative synchronization of local region of tissue to measure intrinsic neuronal activity (Yang et al., 2014a; Liu et al., 2021). Degree of centrality (DoC) is a measure of the mean connections between a voxel as a node and the rest of the brain network (Wang et al., 2017). It represents the level of synchronization in signal fluctuation between the voxel and the rest of the brain. Voxel-mirrored homotopic connectivity (VMHC) indicates the functional connectivity between a voxel and its corresponding voxel in the same location in the contralateral hemisphere (Mancuso et al., 2019). It represents the Pearson's correlation coefficient between the signal fluctuations over a time course of symmetric voxels to show degree of interhemispheric functional connectivity (Zuo et al., 2010).

In this study, we examined whether resting-state fMRI (rsfMRI) analysis of patients with NLE correlates with the seizure onset zones and regions of early and late seizure spread identified using sEEG. Through this analysis, we aimed to see whether noninvasive fMRI imaging techniques can potentially be used to predict patterns of seizure spread, providing insight into the disease process and potentially demarcating areas for intervention without the use of invasive techniques such as sEEG.

## 2. Methods

### 2.1. Subjects

This is a retrospective study examining 8 patients with NLE (ages 25–53, median 35) and 10 healthy controls (ages 22–35, median 27). The patients with NLE all received sEEG after conventional work-up was unable to locate the seizure onset zone. Demographic and clinical characteristics of patients with NLE and controls are displayed in Tables 1, 2, respectively.

**Table 1:** Demographic and clinical information about the patients in the NLE cohort.

**NLE patient number**	**Sex**	**Age at scan**	**Duration**	**Seizure type**	**Surgical intervention**	**Surgical outcome**	**Trialed meds**
1	F	34	12	FIA	VNS then DBS	NSF	CNB, BRV, LEV, CBZ, OXC
2	M	36	1	FIA, FtGTC	DBS	NSF	CNB, CLB, LEV, LMG, LCM, ZNS
3	F	35	16	FA, FIA, FtGTC	Insular resection	SF	BRV, LCM, OXC, LEV, TPM
4	F	53	45	FIA, FtGTC	–	–	LMG, LEV, OXC, TPM
5	F	43	14	FIA	DBS	SF	CNB, GBP, VPA, PHT, LCM, OXC, TPM, PPL, LMG, LEV, PRG, EZG, ESL, PB, TGB, BRV, PSL
6	M	35	17	FA, FIA, FtGTC	LA	NSF	OXC, CNB, LEV
7	M	25	6	FIA, FtGTC	–	–	CNB, LZP, LEV, LMG, OXC, VPA, PHT, ZNS
8	F	30	13	FIA	ATL	SF	LEV, LMG, VPA, ZNS

FA, focal aware; FIA, focal impaired awareness; FtGTC, focal-to-generalized tonic clonic; ATL, anterior temporal lobectomy; DBS, deep brain stimulation; LA, laser ablation; VNS, vagal nerve stimulator; SF, seizure-free; NSF, not-seizure-free; BRV, brivaracetam; CBZ, carbamazepine; CLB, clobazam; CNB, cenobamate; ESL, eslicarbazepine; EZG, ezogabine; GBP, gabapentin; LCM, lacosamide; LEV, levetiracetam; LMG, lamotrigine; LZP, lorazepam; OXC, oxcarbazepine; PB, phenobarbitol; PHT, phenytoin; PPL, perampanel; PRG, pregabalin; PSL, padsevonil; TGB, tiagabine; TPM, topiramate; VPA, valproic acid; ZNS, zonisamide.

**Table 2:** Demographic information about the controls.

**Control number**	**Sex**	**Age at scan**
1	F	23
2	M	26
3	M	22
4	F	24
5	F	28
6	M	35
7	M	23
8	M	30
9	M	30
10	M	31

### 2.2. Image acquisition

All patients underwent rsfMRI scans prior to surgery using a 3.0T Phillips scanner with a 32-channel head coil. rsfMRI images were acquired axially using a single-shot echo planar imaging (EPI) sequence in the same anatomical location prescribed for T1-weighted images. The T1-weighted imaging parameters used were: FOV = 24.0 cm, voxel size = 0.75 x 0.75 x 1.0 mm^3^, matrix size = 320 x 320, TR = 12ms, TE = 6 ms and slice thickness = 1 mm. Resting state imaging parameters were FOV=24.0cm, acquisition matrix size = 68 × 68, acquisition voxel size = 3.5 × 3.5 × 3.5 mm^3^, reconstructed matrix size= 128 × 128, reconstructed voxel size (in-plane resolution) = 1.87 × 1.87 × 3.5 mm^3^ (slice thickness = 3.5 mm), number of slices = 34, TR = 2 s, TE = 25 ms, number of averages = 1 and acquisition time = 12 min (360 volumes). No in-plane acceleration was applied. The reconstructed matrix and voxels were generated through an interpolation step imbedded in the scanner. Participants were instructed to relax, keep their eyes open and think of nothing in particular during resting state scan.

### 2.3. Image processing

Subject MR images were processed through an established preprocessing pipeline for seed-to-voxel and BOLD signal analysis using the FSL MELODIC toolbox (Jenkinson et al., 2012; Sathe et al., 2022). In this pipeline, the functional scans underwent skull-stripping, normalization, smoothing, and MCFLIRT motion correction. The smoothing kernel was FWHM = 4 mm, to approximate double the value of the reconstructed voxel size. The processed functional scans were registered using a linear registration algorithm to each patient's respective T1 image and then to the Montreal Neurological Institute (MNI) 152 standard space with a resampling resolution of 2 mm. Each subject generated two output images, which were filtered_func, the functional data over the time course, and mean_func, the intensity-based image in which each voxel's value represented the average intensity of that voxel over the time course.

The onset zone (OZ), early spread zone (ESZ), and late spread zone (LSZ) were determined for each patient by generating spheres with a 2.5 mm radius around points of involved sEEG contacts, as identified by an epileptologist. The number of sEEG electrodes used was dependent on patient characteristics, seizure semiology, neuropsychiatric evaluation, EEG recordings, PET data, and MRI data. As such, each patient was implanted with a slightly different number of electrodes based on this clinical data, leading to some variations in sEEG resolution. Data on sEEG characteristics is presented in Table 1. The ESZ was defined as the region to which the seizure spread immediately subsequent to onset, prior to any evolution in seizure characteristics. This was generally on the order of milliseconds to a second. The LSZ was defined as the regions surrounding the contacts that were involved once the seizure demonstrated evolution. LSZ regions were identified for three patients for whom we had sufficient data. These regions were segmented in patient T1 space to which the sEEG data was registered for each patient. Volumes were segmented in such a way as to ensure that there were no overlapping volumes between the OZ, ESZ, and LSZ. Information about the location of the OZ, ESZ, and LSZ for each patient with NLE is included in Table 3.

**Table 3:** MNI coordinates and anatomical locations of the OZ, ESZ, and LSZ for each patient with NLE.

**Patient number**	**OZ**	**ESZ**	**LSZ**	**OZ location**	**ESZ location**	**LSZ location**
I	(−29, 5, −25)	(34, −16, −17)	–	L MTL	R MTL	–
2	(−22, −20, −14)	(−21, −3, −17)	–	L MTL	L MTL	–
3	(50, −6, −28)	(48, 2, −10)	(41, 35, −4)	R LTL	R STL	R LFL
4	(−28, −19, −18)	(−29, −30, −8)	(−29, −22, −18)	L MTL	L MTL	L MTL
5	(31, −15, −22)	(−28, −17, −18)	–	R MTL	L MTL	–
6	(−26, −11, −17)	(−27, −1, −23)	–	L MTL	L MTL	–
7	(−25, −11, −25)	(−27, −3, −23)	–	L MTL	L MTL	–
8	(−27, −18, −18)	(−10, −19, 31)	(25, −19, 19)	L MTL	L MFL	R putamen

L, left side; R, right side; MTL, medial temporal lobe; LTL, lateral temporal lobe; STL, superior temporal lobe; MFL, medial frontal lobe; LFL, lateral frontal lobe.

Subject T1 and mean_func scans were registered to MNI152 space using the MIRTK registration algorithm. Subject 4D filtered_func scans were transformed to the same space using the transformation matrix from the mean_func registration. OZ, ESZ, and LSZ segmentations were transformed to MNI152 space using the transformation matrix from the T1 registration.

For each NLE patient, amplitude synchronization was applied using the OZ as a seed to find correlations from the seed to every voxel in the brain based on similarity of BOLD fluctuations (Nickerson et al., 2017). The proportions of both positively and negatively significantly correlated voxels (z > 2.5 or z < −2.5) in the volume defined by the ESZ and LSZ were calculated for each NLE patient and added together to find the total proportion of significantly correlated voxels.

The OZ from each NLE patient was used in each control's functional scan to generate statistical maps (z-maps) via seed-to-voxel analysis. The spread zones of each patient were overlaid onto each control's generated z-map using the region corresponding to the OZ from the same patient to calculate the proportion of significantly correlated voxels. As such, regions corresponding to the OZ and spread zones for each NLE patient were used to generate z-maps and voxel proportion data for that patient and all 10 controls.

Data was also preprocessed and analyzed for FC metrics using the Data Processing & Analysis for Resting-State Brain Imaging (DPABI) software (Yan et al., 2016). T1 images were skull stripped and co-registered to the functional images. The functional images had 360 time points and a TR of 2 s. Images were normalized by DARTEL to MNI space. The Friston 24-parameter model was used to correct for head motion artifacts. White matter, CSF, and global signal artifacts were regressed as nuisance covariates.

**Figure 1:**
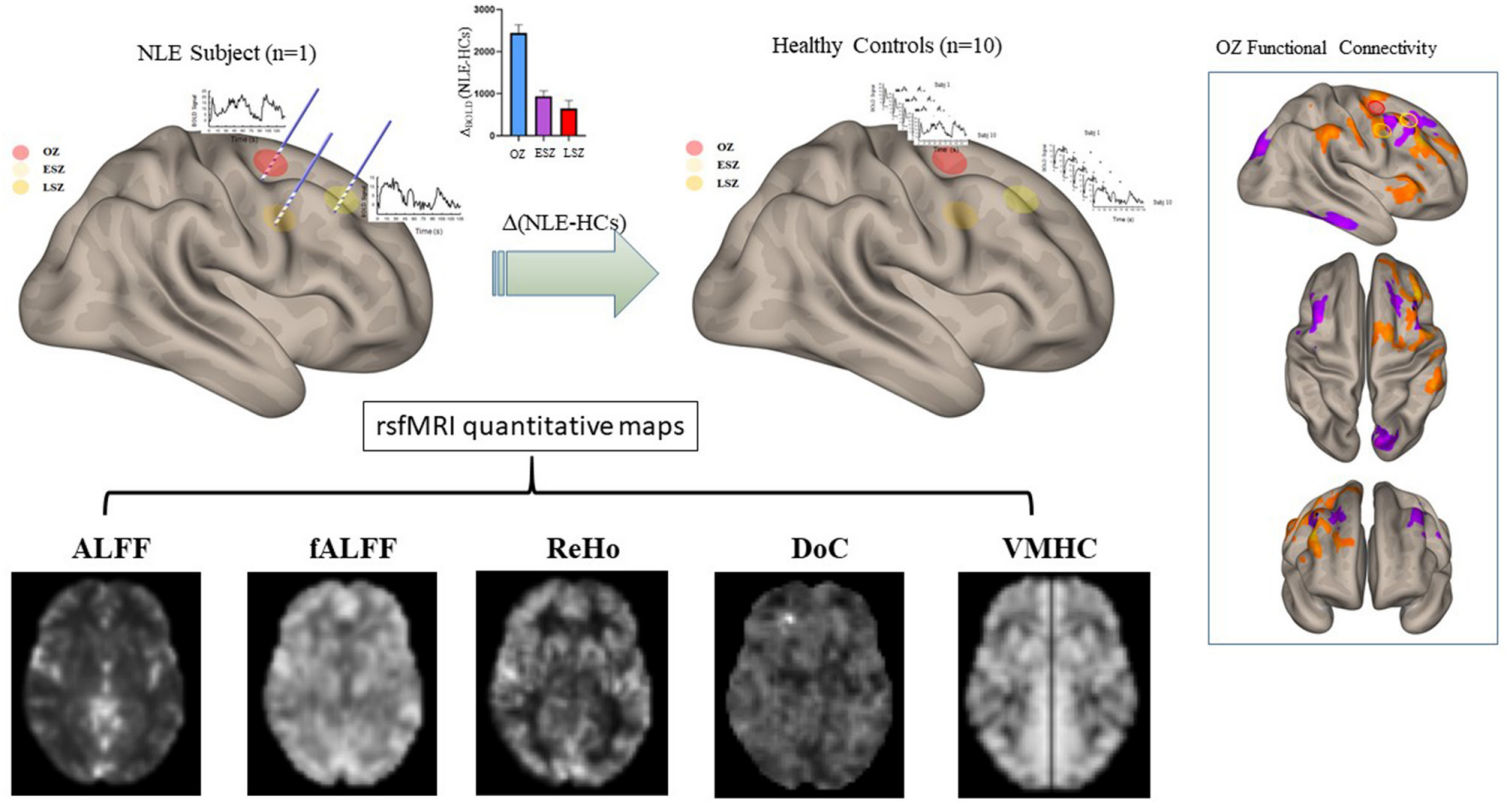
Representation of the processing of the FC maps in patients and controls using one example patient with NLE. The OZ and ESZ were calculated around sEEG contacts in patients with NLE. For each patient with NLE, these same regions were overlaid onto control FC maps to calculate the values in analogous regions. All five FC metrics were calculated and subtracted, NLE minus controls, using the regions generated by that specific patient with NLE. This set of 10 comparisons for each metric, one comparison for each control, was generated for each patient with NLE, leading to a total of 80 comparisons for each metric.

Five functional connectivity measures were calculated for each patient and control using the same sets of segmentations generated using DPABI. The calculated measures were ALFF, fALFF, ReHo, DoC, and VMHC. The frequency band used to calculate ALFF and fALFF was 0.01 to 0.1 Hz. ReHo was calculated used a cluster size of 27 voxels. In all, 8 total sets of NLE data and 80 sets of control data were generated for each of these different measures, as each NLE patient was compared to all 10 controls. This process is depicted in Figure 1.

### 2.4. Statistical analysis

Analysis of seed-to-voxel FC data was performed on an individual subject and group basis. For individual analysis, the OZ-to-ESZ and LSZ FC values that were calculated for the segmentations overlaid onto each control's segmentations were compared to the FC value seen in the NLE subject. A one-sample Wilcoxon test was used to compare the control data to the NLE subject FC value. The proportion of individual patients displaying significant differences from the control means was determined. For group analysis, the FC values between the OZ and the ESZ and LSZ for each NLE subject were grouped and compared to the control FC values for each control using each NLE patient's segmentations using a Mann-Whitney test for nonparametric distribution.

The five FC metrics were each calculated by subtracting the control subject's FC metric value from the data of the respective patient with NLE to determine the difference between control and NLE values. A general linear model was generated with Bonferroni correction using age at scan of controls and patients with NLE as covariates. This was used to create 95% confidence intervals for each metric in the OZ and ESZ to test for difference from zero. Difference values in the OZ and ESZ for each metric were also directly compared using a general linear model using age as a covariate. Bonferroni correction was applied to the general linear model for correction for multiple comparisons.

All data are reported with an alpha of 0.05.

## 3. Results

### 3.1. Functional connectivity differs between seizure-related zones in patients with NLE

**Figure 2:**
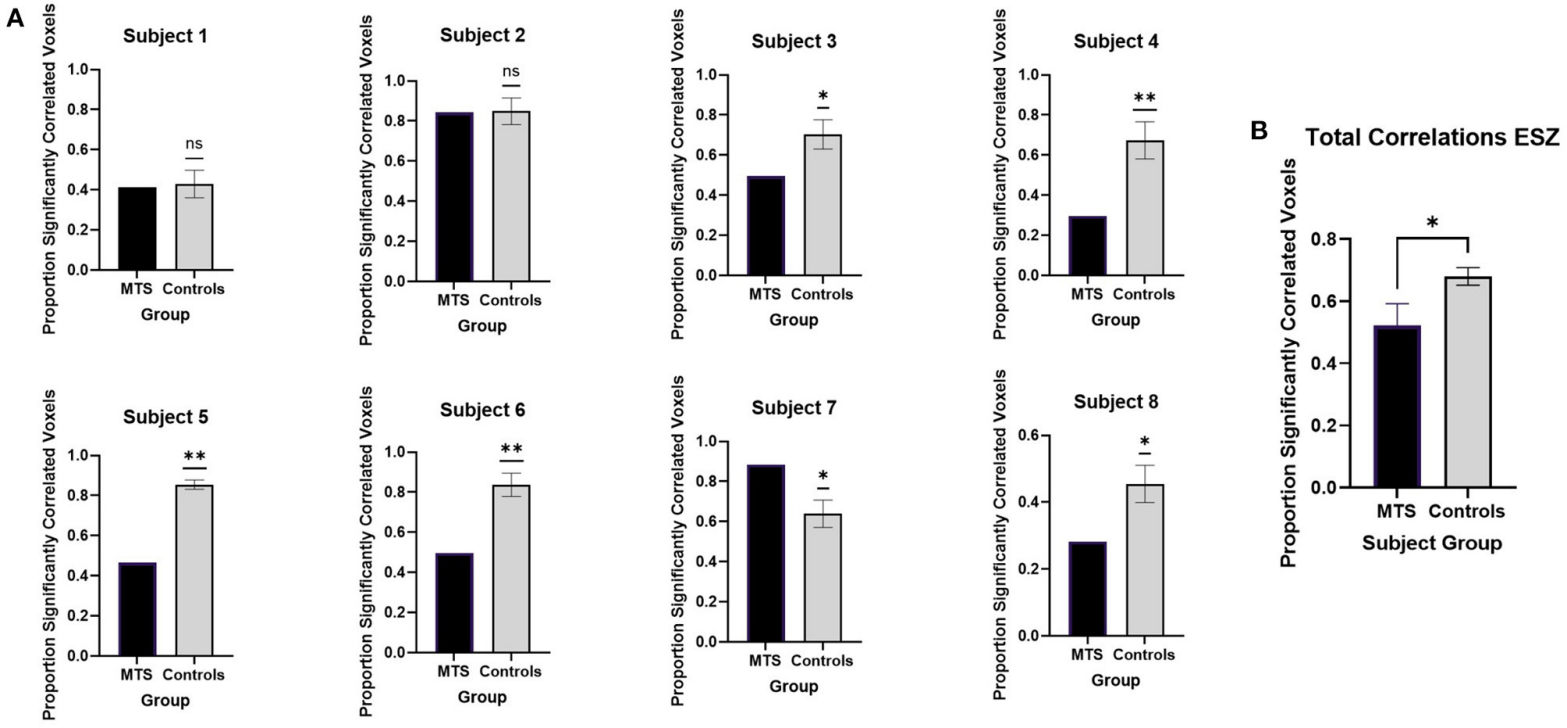
Seed-to-voxel analysis of functional connectivity between the OZ and ESZ with **(A)** individual analysis and **(B)** group analysis. Significant differences between patients with NLE and controls are marked with “*” while nonsignificant differences are marked with “ns.” All significant values are reported with *p* < 0.05.

When analyzed on an individual basis, we found that 5/8 patients with NLE (subjects 3, 4, 5, 6, and 8) showed decreased correlations from the OZ to the ESZ compared to controls (Figure 2A). Only 1 patient with NLE out of 8 (subject 7) showed an increased correlation. Group analysis showed patients with NLE had lower overall connectivity between the OZ and the ESZ compared to controls (Figure 2B). As only 3/8 patients had LSZ data, the data was not included in this analysis.

### 3.2. Functional metrics are altered in seizure-related zones in patients with NLE

**Figure 3:**
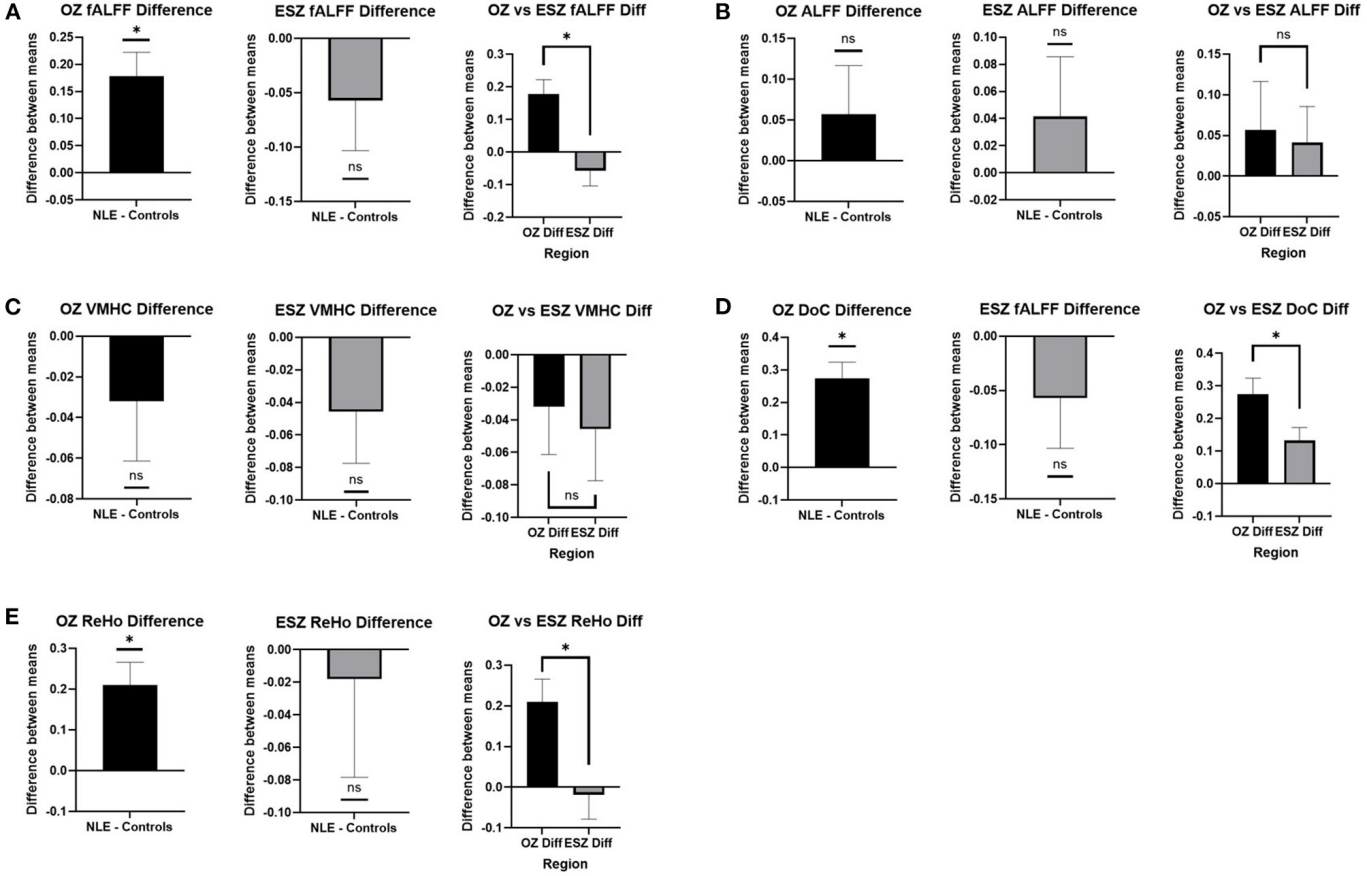
FC metric differences between patients with NLE and controls in the OZ and ESZ and between the OZ and ESZ in **(A)** fALFF, **(B)** ALFF, **(C)** VMHC, **(D)** DoC, and **(E)** ReHo. Mean difference between patients with NLE and controls are shown in the OZ and the ESZ. These values are compared to zero and to each other. Significant differences are shown as “*” while non-significant differences are shown as “ns.” All significant values are reported with *p* < 0.05.

FC metric analysis revealed differences between patients with NLE and controls. The difference between patients with NLE and controls in the OZ was significantly greater than zero for fALFF (Figure 3A), DoC (Figure 3D), and ReHo (Figure 3E), while it was nonsignificant for ALFF (Figure 3B) and VMHC (Figure 3C). Meanwhile, the difference in the ESZ was only significantly greater than zero in NLE when looking at DoC; the other metrics showed no significant difference from zero. Comparison of the difference in the OZ to that in the ESZ showed a significantly higher value in the OZ when analyzing fALFF, DoC, and ReHo, while ALFF and VMHC showed no significant differences between the two regions.

### 3.3. Patient clinical management

Six of the 8 patients received surgical intervention for their refractory epilepsy. Three patients received DBS, of which one patient had failed VNS therapy prior. One patient received insular resection, one received laser ablation to the mesial temporal region, and one received anterior temporal lobectomy. Three patients remained seizure-free within 12 months after surgical intervention, while three patients did not. Information about the interventions received by the patients and subsequent clinical outcomes is listed in Table 1.

### 3.4. Histopathological analysis of resected tissue

Two patients received resective surgery during which tissue samples were obtained for histopathological analysis. Patient 3, who received insular resection, showed tissue with evidence of reactive gliosis. Patient 8, who received anterior temporal lobectomy, showed tissue with mild cortical gliosis without any specific pathological changes in the hippocampus or amygdala.

## 4. Discussion

Our results demonstrated decreased overall correlations between the OZ and spread zones in patients with NLE when looking at BOLD signal fluctuation via rsfMRI, indicating decreased functional connectivity between these seizure-related regions. This was contrary to our expectations that regions of seizure spread would show increased synchronization with the OZ, as prior studies with stereotactic and intracerebral EEG have shown increased effective connectivity between regions of seizure onset and spread (Lega et al., 2015; Parker et al., 2018). However, Bettus et al. (2011) also showed opposite findings when comparing connectivity derived from analysis intracerebral EEG and fMRI. These findings may reflect differences in the hemodynamic function underlying BOLD signal compared to the electrophysiological mechanisms determining EEG data, as BOLD signal shows a delayed time course to neuronal activation while EEG electrophysiological data measures real-time fluctuations (Gupta et al., 2020). In addition, fMRI generally measures low-frequency fluctuations, and has been shown to find increased temporal correlations in low-frequency bands (Biswal et al., 1995; Salvador et al., 2005). In contrast, sEEG recordings can detect spectral correlations at much higher frequencies (Bettus et al., 2011). As such, these negatively correlated findings may also reflect measurement of separate processes occurring at different frequencies that behave differently from each other but are both influenced by the same pathological processes in epilepsy.

Prior literature has also shown decreased functional connectivity as detected by fMRI BOLD signal in focal epilepsy. Pittau et al. (2012b) showed decreased connectivity involving the default mode network and the lesional-side hippocampus and amygdala in patients with mesial temporal sclerosis (Pittau et al., 2012b). Although our results showed selective decreases in FC from the OZ to spread zones rather than decreases in global network connectivity, all of these findings may reflect functional disconnection and disruption of the focal onset region to other brain regions.

We saw increased fALFF signal in the OZ, indicating increased neuronal activity in the epileptogenic region in patients with NLE. This may indicate pathologic hyperactivity involving epileptogenic networks causing an increase in seizure propensity in these patients. This increase in neuronal activity was seen most markedly in the OZ while it was not seen in the ESZ, indicating that tissue that is pathologically activated early in the seizure course is the most hyperactive and abnormal of the neuronal tissue involved in the seizure network. Prior studies have shown low-frequency signal analysis to reflect spontaneous activity in the brain (Yang et al., 2007; Zang et al., 2007; Zhang et al., 2010). Zhang et al. (2010) suggested that increases in low-frequency signal amplitudes represent interictal epileptic activity. Interestingly, prior literature examining neuronal glucose metabolism via PET studies has shown focal hypometabolism in regions adjacent to seizure-related areas. Donaire et al. (2013) demonstrated that, while the epileptogenic zone appears to be hypometabolic compared to healthy neural tissue, there is a focal increase in neural activity around those same regions during or just prior to interictal epileptiform discharges. Juhász et al. (2000) and Alkonyi et al. (2009) found that seizure activity was increased near border zones between areas of hypometabolism and normal metabolism. As such, the increase in neuronal activity seen in the OZ may reflect spikes in metabolic border regions. Co-analysis using fMRI and metabolic studies may provide a noninvasive method to localize seizure-related regions to areas with discordant BOLD and PET findings.

We also found alterations in other functional metrics in the OZ and the ESZ in NLE compared to controls. Interestingly, DoC was a metric in which significant differences were seen both in the OZ and the ESZ. The DoC metric represents the degree of connectivity between a voxel and the rest of the brain, indicating in this case that epileptogenic regions are more highly connected with other brain regions in patients with the disease compared to those same regions in healthy tissue. Prior literature has also found alterations in DoC in different regions of the brain in epilepsy patients, although these alterations include both increases and decreases in global connectivity in various seizure-involved regions. Piper et al. (2021) studied pediatric patients with focal epilepsy and found increased DoC in the thalamus, a region known to be involved in seizure spread in different forms of epilepsy (Bertram et al., 2001; Piper et al., 2021). Gao et al. (2022) found increased DoC in frontal cortical structures in patients with temporal lobe epilepsy but decreased DoC in temporal regions, including the hippocampus. These findings suggest that pathological changes in connectivity in seizure-involved regions may facilitate the spread of seizure impulses and mark abnormal tissue, although the literature does not fully agree on the direction of the alterations. The increase in DoC found in our study appears to be contradictory to our findings of decreased synchronization between the OZ and spread zones. However, the sEEG leads used to sample the signals used to construct the seed regions were purely cortical and did not sample subcortical regions such as the thalamus. As such, our findings from the amplitude synchronization analysis represent disruptions in cortical connectivity in patients with NLE, while the increases in DoC we found in patients with NLE represent increases in global connectivity, possibly through subcortical relays involving structures such as the thalamus.

We also found that ReHo was significantly higher in patients with NLE in the OZ. As ReHo represents local synchronization of BOLD fluctuations, these results indicate greater synchronization of neuronal activity in the epileptogenic region. Mankinen et al. (2011) found increased ReHo in regions including the right mesial temporal area in pediatric patients with non-lesional temporal lobe epilepsy. Liu et al. (2021) also found increased ReHo in the bilateral prefrontal gyri in patients with focal frontal lobe epilepsy, a region believed to be an epileptogenic focus in the disease. Bettus et al. (2008) found increases in local FC near the epileptogenic zone through EEG studies. Interestingly, Englot et al. (2015) found that patients with focal epilepsy who demonstrated increased regional FC within the presumed epileptogenic zone through magnetoencephalographic studies were more likely to respond well to resective surgery. The authors in that study suggested that increases in local FC may delineate the true epileptogenic zone, thereby providing a potential biomarker to target for resective surgery (Englot et al., 2015). The increased degree of ReHo seen selectively in the OZ in our study agree with this idea. Ridley et al. (2017) studied within-region FC in seizure-generating and seizure-associated regions via simultaneous sEEG-fMRI recordings and found an increase in intrinsic BOLD-FC within the primary irritative zone (Ridley et al., 2017). Our measures of intrinsic, local FC agree with these findings, showing that the large increase in local, intra-regional FC in the OZ may reflect a pathological ability of the tissue to generate and sustain abnormal electrical activity. Additionally, they found that the inter-modality agreement in FC measurement was high in regions not involved in seizures, but was differentially altered in the onset and spread zones, in which electrophysiological and BOLD data showed an inverse relationship, as seen in other studies comparing the modalities (Bettus et al., 2011; Ridley et al., 2017). However, they did not examine FC between seizure-related zones to study the alterations that may underlie seizure propagation, nor did they compare FC values in patients with epilepsy to those of healthy controls.

The ReHo findings combined with the fALFF and DoC results show increases in both local and global synchronization in the OZ and greater global connectivity in the ESZ. When combined with the decreases in connectivity seen between the OZ and ESZ, our results suggest cortical disassociation with overall increased global connectivity, indicating abnormalities in cortical seizure-related areas, but also in other regions, likely subcortical, compared to healthy neural tissue.

Our findings showed no differences in VMHC in seizure-related areas in patients with NLE compared to healthy controls. Prior studies have found alterations in VMHC in various brain regions including parts of the frontal, temporal, and parietal cortices (Yang et al., 2014b; Liu et al., 2016). However, these regions do not necessarily represent proximal areas of seizure onset or spread. The lack of changes seen in our analysis may be due to the narrow focus of studying VMHC in the OZ and ESZ rather than through a comprehensive analysis of all brain regions.

Clinical outcomes after surgical intervention varied between the patients in this study, with three patients still experiencing seizures within 12 months of receiving surgery. Two of the patients who were not seizure-free had received DBS implantation to control their seizures. A review paper found that studies consistently show that DBS often does not leave a patient seizure-free after implantation, but does lead to a robust decrease in seizure frequency (Zangiabadi et al., 2019). A RCT studying the effectiveness of ANT-DBS for refractory epilepsy showed a 41% reduction in seizure frequency compared to baseline, with only 1.8% of the treatment group achieving seizure-freedom within months 4–13, which was after stimulation was started (Fisher et al., 2010). Due to the low sample size and the focus of this paper, we did not stratify patients into the degree of seizure reduction, and were unable to perform any significant statistical analysis comparing such groups.

The third patient who was not seizure-free received laser ablation to the MTL. It is possible that the surgical target determined via sEEG did not accurately localize the OZ in this case. However, prior literature has shown that volume of ablation in the epileptogenic region may not necessarily impact seizure outcomes (Kang et al., 2016; Youngerman et al., 2018). A paper examining laser trajectory found that treatment failure may be attributable to a laser course that may not adequately ablate portions of the hippocampal head, which is associated with seizure recurrence after surgery (Jermakowicz et al., 2017). Other possibilities for treatment failure after laser ablation may include anatomical difficulties in laser targeting and the presence of heat sinks such as blood or CSF that may lead to inadequate ablation despite accurate localization (Kang et al., 2016). Additionally, patients with mesial temporal lobe epilepsy without evidence of MTS are more likely to show treatment failure (Téllez-Zenteno and Hernández-Ronquillo, 2012). Unfortunately, we do not have a histopathological specimen from the patient who received laser ablation, so we are unable to clarify this question. Future studies with larger sample sizes examining seizure outcomes, potentially with multiple categories related to degree of reduction of seizure frequency, may be able to elucidate whether the methods we explored in this study may have a beneficial impact on patient surgical outcomes.

The histological specimens obtained from both of the patients who received resective surgery showed some evidence of reactive gliosis but no specific pathological abnormalities. Regardless of any changes in the tissue, the lack of an identifiable lesion on standard clinical work-up and imaging properly categorizes these patients as having had NLE.

Limitations in this study include a lower sample size of our patient cohort, especially when analyzing LSZ data. The low statistical power for the LSZ makes it difficult to find alterations in long-term seizure spread regions. Additionally, while the NLE and control cohorts were matched with respect to age to the best of our abilities, there was still a difference in the median age between the two groups (35 versus 27 years). However, we used age as a covariate in our general linear model when performing group analysis to minimize confounding effects. Another limitation is that we did not include the type of seizure experienced by the patients in the study beyond NLE status. Future work could account for seizure class, such as generalized tonic-clonic or focal subtypes, which may add more dimension to the analysis. This would also require a larger sample size for sufficient analytical power. Furthermore, the segmentations around involved sEEG contacts used to define the OZ, ESZ, and LSZ were empirically defined using a 2.5 mm radius. The volume around the contact that was used in FC analysis may impact the overall FC results, but only one such radius length was used in this study. Future studies may benefit from analyzing a variety of radius lengths to assess whether results are consistent over a multitude of selected radii. Other future directions for this analysis include examining functional connectivity alterations present between regions of seizure onset and spread by simultaneously studying electrophysiology and fMRI via sEEG-fMRI or similar modalities. This may reveal direct correlations between functional and stereotactic data in detecting inter-region connectivity.

## 5. Conclusion

This study shows the efficacy of rsfMRI in identifying alterations in connectivity between areas of seizure onset and spread in patients with NLE. Although the functional connectivity detected via this technique was the opposite of sEEG data, these findings agree with past literature and likely represent alterations stemming from the same pathologic processes.

We have also shown differences in various FC metrics and activation in abnormal seizure regions in patients with NLE. These differences in FC metrics between patients with NLE and controls indicate functional changes in the disease related to seizure activity. The differences in functional metrics between the OZ and ESZ again support the notion that the OZ is more pathologically altered compared to regions where the seizure spreads to at a later time course.

Detection of alterations in regions involved in seizure onset and spread establishes resting state fMRI FC analysis as a modality to delineate abnormal, epileptogenic tissue via noninvasive imaging techniques. Furthermore, finding alterations in different metrics provides insight into the pathophysiology of the disease and may help uncover further information on the disease process. Combining these functional modalities with other noninvasive techniques such as PET scans may provide even more utility in localizing lesions.

Future studies may examine alterations in FC metrics in patients based on differences in seizure classification. Prospective studies may be performed to look at clinical outcomes based on concordance of surgical targets with regions identified as seizure-related.

## Data availability statement

The original contributions presented in the study are included in the article/Supplementary material, further inquiries can be directed to the corresponding author.

## Ethics statement

The studies involving human participants were reviewed and approved by Thomas Jefferson University Institutional Review Board. The patients/participants provided their written informed consent to participate in this study.

## Author contributions

AVS performed data processing and analysis and wrote the main text of the manuscript. CM and MA helped edit the main text. AVS and MA created the figures included in the manuscript. All authors reviewed and approved of the manuscript prior to publication.
